# Glossary

**Published:** 1995

Adenylyl cyclase: An *enzyme* used by cells, including *neurons*, to relay signals from the exterior to the interior of the cell.
Allele: One of two or more variants of a *gene*. Different alleles for a gene serve the same function (e.g., code for a protein that affects a person’s eye color) but may result in different *phenotypes* (e.g., blue eyes or brown eyes).
Amino acids: The building blocks of *proteins*. Some amino acids also serve as *neurotransmitters*.
Antisense oligonucleotide: A short string of *nucleotides* that can bond to *messenger RNA (mRNA)* and block the process of *gene expression*.
Association analysis: A technique to identify *gene* variants (i.e., *alleles*) that might predispose a person to a disease but that are not required for disease development.
Candidate gene: A *gene* that has been implicated in causing or contributing to the development of a particular disease.
Centimorgan (cM): The morgan (M) is the standard unit of measure of the relative distances between *genes* on a *chromosome;* for working purposes, the centimorgan (0.01 M) is used.
Chromosomes: Threadlike molecules in the cell nucleus that consist of *DNA* and *protein* and contain most of the cell’s *genes*. Humans have 46 chromosomes arranged in 23 pairs.
Congenic strain: A strain of organisms, such as mice, created by transferring through specific breeding strategies a small segment of a *chromosome* from one strain into another *inbred strain*. The only difference between the congenic and the inbred strain is this small chromosome segment.
Crossing over: A reaction between the two partners of a *chromosome* pair during which sections of the *chromosomes* break off and are exchanged between the partners. Crossing over occurs during *meiosis*.
Cystic fibrosis: A genetic, metabolic disorder affecting primarily the lungs and airways, characterized by the production of thick mucous; it usually is recognized in early childhood and frequently causes death during young adulthood.
Cytoplasm: The part of the cell outside the nucleus.
DNA: The abbreviation for deoxyribonucleic acid, the molecule that encodes the genetic information in all organisms except some viruses. DNA molecules usually consist of two strings of *nucleotides* (see figure). DNA is a component of *chromosomes*.
Dopamine: A *monoamine neurotransmitter* that is important in the parts of the brain that regulate movement, mood, and the rewarding effects of alcohol and other drugs.
Electrophoresis: A technique for separating and purifying molecules of different size according to the relative distance they travel under the influence of an electric current.
Enzyme: A *protein* that catalyzes (i.e., directs and accelerates) chemical reactions in the cell.
Gamete: Any mature *germ cell*.
Gene: A string of *nucleotides* that directs the synthesis of a *protein*.
Gene expression: The process of converting the genetic information encoded in the *DNA* into the final *gene* product (i.e., a *protein*).
Genome: The sum of all *genes* in an organism.
Genotype: The genetic makeup of an individual organism.
Germ cell: A cell responsible for reproduction (i.e., the sperm cells in males and the eggs in females) or its precursors. Germ cells contain only half the number of *chromosomes* as *somatic cells*.
Heterozygous: Having different *alleles* of a *gene* at corresponding *loci* on the two partners of a *chromosome* pair.
Homozygous: Having identical *alleles* of a gene at corresponding *loci* on the two partners of a *chromosome* pair.
Huntington’s disease: A degenerative, genetic disorder of the central nervous system, usually beginning in young to middle age, characterized by progressive mental deterioration and abnormal involuntary muscular movements.
Inbred strain: A virtually genetically identical group of organisms derived by inbreeding among a limited number of ancestors. An inbred strain of mice is like a population of identical twins.
Independent assortment: The principle that during *meiosis* the two copies of each *gene* are distributed to the *germ cells* independently of the distribution of other genes. Independent assortment is limited by the *linkage* of genes that are located close to each other on the same *chromosome* and thus tend to be inherited together. (See figure 2, p. 223.)
Isoenzyme: An *enzyme* performing the same function as another enzyme but having a different *amino acid* composition. Isoenzymes can differ in their *kinetic properties*.
Kinetic properties: The characteristics of *enzymes* and other chemicals that describe the speed and effectiveness with which they catalyze a chemical reaction.
Knockout mice: Mice in which a *gene* has been deleted or inactivated in both the *somatic* and the *germ cells* so that the animals produce no functional gene product.
Linkage: The tendency for *genes* that are located close to each other on the same *chromosome* to be inherited together.
Linkage analysis: Research technique to identify *gene* variants that are necessary or sufficient to cause a disorder (compare with *association analysis*).
Locus, loci: A specific location(s) on a *chromosome*.
Lymphocyte: A white blood cell that is important in the body’s immune response. Because they contain some *proteins* also found in *neurons*, lymphocytes serve as more easily accessible models of some neural functions.
Mapping: The process of determining the position of a *gene* on the *chromosome* relative to other genes.
Marker: A characteristic by which a cell or molecule can be recognized or identified. Genetic markers consist of specific *nucleotide* patterns.
Meiosis: The specialized process of cell division that creates *germ cells* (i.e., sperm cells and eggs). (See figure 2, p. 223.)
Messenger RNA (mRNA): A type of *RNA* molecule that carries the information copied from a *gene* and serves as a template for the production of *proteins*.
Mitochondrion: An *organelle* within a cell that generates most of the cell’s energy.
Monoamine neurotransmitters: A category of *neurotransmitters* that transmit signals between *neurons*. The monoamine neurotransmitters include norepinephrine, *dopamine*, and *serotonin*.
Monoamine oxidase (MAO): The *enzyme* that breaks down *monoamine neurotransmitters*.
Mutation: A change, deletion, or rearrangement in the *DNA* sequence that may lead to the synthesis of an altered *protein* or to a totally inactive *gene* incapable of producing a protein.
Neuron: A nerve cell.
Neurotransmitter: A chemical released by *neurons* that excites or inhibits other nerve, muscle, or gland cells.
Nucleotide: The building block of *DNA* or *RNA*. Each nucleotide consists of a sugar component, a phosphate group, and an organic base. Four organic bases exist in *DNA* (adenine, cytosine, guanine, and thymine) and in *RNA* (adenine, cytosine, guanine, and uracil). Specific strings of DNA nucleotides make up *genes*.
Oligonucleotide: A molecule made up of a small number of *nucleotides*, typically fewer than 20. Researchers use these in genetic experiments (e.g., to link fragments of *DNA* together).
Organelle: Microscopic structures in a cell that have specialized functions (e.g., *mitochondria* and the nucleus).
Phenotype: The observable properties, traits, or physical appearance of an organism resulting from the interaction of the *genotype* with environmental factors.
Platelet: A disklike blood cell involved in blood clotting. Because they contain some *proteins* also found in *neurons*, platelets frequently serve as more easily accessible models of some neural functions.
Polymerase chain reaction (PCR): An enzymatic technique for producing multiple copies of a specific piece of *DNA*.
Polymorphism: For a specific *gene*, the presence of two or more gene variants (i.e., *alleles)* in a population.
Primer: A molecule that initiates the synthesis of a larger molecule. For example, a short, synthetic piece of *DNA* serves as a primer to initiate a *polymerase chain reaction*.
Probe: A piece of *DNA* used to locate another piece of DNA. The probe, which can bond with the DNA of interest, usually is made radioactive or fluorescent so that it can be detected easily.
Protein: The product of the genetic information encoded in a *gene*. Proteins are made up of *amino acids* whose order is dictated by the gene’s *nucleotide* sequence. *Enzymes* are one type of protein.
Quantitative trait: A trait, or characteristic, that is determined by more than one *gene* and which exists in many different degrees (i.e., is distributed continuously) within a population. Body height is an example of a quantitative trait.
Receptor: A *protein* usually found on the surface of a cell that binds to a specific chemical messenger, such as a *neurotransmitter*.
Recombinant inbred (RI) strains: A set of animal strains derived by inbreeding the offspring from two parent strains. Each strain in an RI set has a unique combination of *genes* from the parent strains, and all animals in a single strain have the same gene combination. RI strains aid researchers in determining the genes that affect certain traits.
Recombination: The formation of new combinations and arrangements of *genes* during *meiosis;* recombination is achieved by *crossing over, independent assortment*, and *segregation*.
Restriction enzymes: A group of *enzymes* isolated from bacteria that cut *DNA* molecules at specific sites characterized by certain *nucleotide* sequences.
Restriction fragment length polymorphism (RFLP): A variation among individual organisms in the size and number of *DNA* fragments generated by the actions of *restriction enzymes*. These variations can be detected by the differences in the distribution of *DNA* fragments during *electrophoresis*.
RNA: The abbreviation for ribonucleic acid, a *DNA*-like molecule. Different kinds of RNA exist that play specific roles in the process of *gene expression*.
Segregation: The principle that the two partners of a *chromosome* pair are separated during *meiosis* and distributed randomly to the *germ cells*. Each germ cell has an equal chance of receiving either chromosome.
Sequencing: The process of determining the sequence of *nucleotides* in a piece of *DNA* or of *amino acids* in a *protein*.
Serotonin: A *monoamine neurotransmitter* that affects mood, consummatory behaviors, and the development of tolerance to alcohol.
Somatic cells: All cells of an organism other than the *germ cells*.
Transfer RNA (tRNA): A group of *RNA* molecules that transport specific *amino acids* to the site of *protein* synthesis within the cell.

**Figure:**
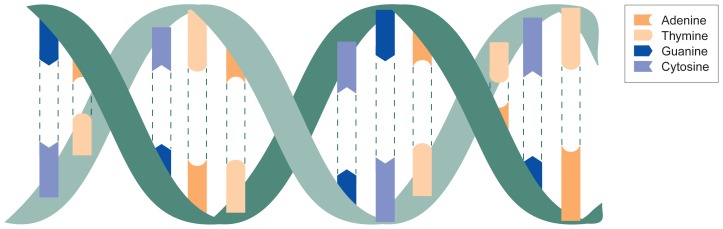
DNA includes four types of nucleotides: adenine, thymine, guanine, and cytosine. Adenine always bonds with thymine, and guanine always bonds with cytosine. These two combinations form the double-stranded DNA molecule.

